# Elevated symptoms of muscle dysmorphia and disordered eating among male gym-goers in Riyadh: a cross-sectional screening study

**DOI:** 10.1186/s40337-026-01556-3

**Published:** 2026-02-24

**Authors:** Khaldoun Ibrahim Marwa, Nawaf Salah Ayad Mohamed, Hassan Mohammed Abdu, Abdulrahman Abduljabbar Alsarari, Shaden Ibrahim Alsenidi, Nasser Eid Alotaibi, Mohammed Adel Alrehaili, Rayan Saleh Almughyir, Noof K. Binashikhbubkr, Anas A. Abdulkader

**Affiliations:** 1 AlMaarefa University, Riyadh, Saudi Arabia; 2 College of Medicine, AlMaarefa University, P.O.Box 71666, Riyadh, 11597 Saudi Arabia; 3 College of Medicine, Prince Sattam bin Abdulaziz University, Alkharj, Saudi Arabia; 4 College of Medicine, Al Jouf University, Sakakah, Saudi Arabia; 5 College of Medicine and Health Science, Hadhramout University, Hadhramout, Yemen

**Keywords:** Muscle dysmorphia, Body image, Eating disorders, Gym, Saudi Arabia, MDDI, PHQ-ED

## Abstract

**Background:**

Muscle dysmorphia (MD) refers to distressing concerns about being insufficiently muscular and is increasingly reported among gym-going men. Evidence from Saudi Arabia is limited. This study estimated the proportion of gym-going men in Riyadh who screened positive for elevated MD symptomatology and examined associations with disordered eating symptoms and sociodemographic factors.

**Methods:**

A cross-sectional survey was conducted among male gym-goers in Riyadh. Elevated MD symptomatology was assessed using the Muscle Dysmorphic Disorder Inventory (MDDI), and disordered eating symptoms were screened using the Patient Health Questionnaire–Eating Disorders Module (PHQ-ED). Group differences were examined using appropriate bivariate tests. A stepwise binary logistic regression model was used to identify predictors of screening-positive status for elevated MD symptomatology (MDDI ≥ 39).

**Results:**

Of 303 participants, 49.8% screened positive for elevated MD symptomatology (MDDI ≥ 39). Screening-positive PHQ-ED status was associated with screening-positive MDDI status (Fisher’s exact test, *p* = 0.030). In the final stepwise multivariable model, marital status and graduate-level education were associated with screening-positive MDDI status. Body mass index (BMI) was not retained in the final stepwise model, and PHQ-ED status could not be evaluated in the multivariable model due to sparse data/complete separation (non-estimable odds ratios).

**Conclusions:**

Nearly half of the surveyed gym-going men screened positive for elevated MD symptomatology, indicating a high symptom burden in this selected sample. These findings should not be interpreted as diagnostic prevalence. Screening-based approaches in gym settings may help identify individuals who report high distress or interference and may benefit from further clinical assessment. Larger studies using probability sampling and structured diagnostic interviews are needed to estimate diagnostic prevalence and clarify associated factors.

**Supplementary Information:**

The online version contains supplementary material available at 10.1186/s40337-026-01556-3.

## Abstract (plain language summary)

Many men worry about not being “muscular enough.” For some people, these worries can become intense and interfere with daily life; this pattern of severe muscularity-related distress is sometimes referred to as muscle dysmorphia. We conducted a survey to understand these concerns among men who attend gyms in Riyadh, Saudi Arabia. We used a screening questionnaire with 303 men. Nearly half of the participants scored above the questionnaire’s screening threshold for elevated symptoms related to muscle dysmorphia. A high score does not constitute a formal medical diagnosis, but it may indicate greater distress or interference that could benefit from further assessment. We also found that screening positive for muscle-related concerns was linked with screening positive for eating-related concerns. Interestingly, men who were married or divorced were more likely to screen positive, and men with higher levels of education (such as a Master’s or Doctor of Philosophy (PhD)) were also more likely to report elevated symptoms. A person’s measured body size or weight was not linked to these concerns, suggesting the issue relates more to body perception than to actual size. These findings support the value of awareness and clear referral pathways in gym settings for individuals reporting high distress or impairment.

**Supplementary Information:**

The online version contains supplementary material available at 10.1186/s40337-026-01556-3.

## Background

 Body image concerns related to muscularity exist on a continuum, ranging from normative dissatisfaction to severe psychological distress and functional interference. At the severe end of this spectrum, muscle dysmorphia (MD) is described in the Diagnostic and Statistical Manual of Mental Disorders, Fifth Edition (DSM-5) as a subtype of body dysmorphic disorder (BDD) [1]. First identified among bodybuilders [2], MD refers to individuals who are preoccupied with the belief that they are insufficiently large and muscular. Their daily lives may become dominated by activities aimed at increasing muscularity, such as intensive weightlifting, restrictive diets, and substance use [3]. Modern societies and cultures, embedded in a technological and social media context, increasingly promote physical perfection and hyper-muscular ideals [4]. While both men and women can experience these concerns, research indicates that males typically report higher levels of muscularity-oriented symptomatology than females [5].

In particular, men commonly report dissatisfaction with their perceived lack of muscle, which may be accompanied by negative emotions (e.g., worry, anxiety, and upset) and behaviors aimed at altering muscular shape [6]. Dysfunctional eating patterns, alongside psychological traits such as perfectionism, low self-esteem, and social anxiety, may contribute to the development and maintenance of these symptom patterns [7]. Reported proportions of screening-positive individuals for elevated MD symptomatology vary widely worldwide, with at-risk estimates ranging from 3.6% to 58.3%, depending on the screening instrument and population studied [8]. However, no studies have documented screening-positive proportions for elevated MD symptomatology in Saudi Arabia yet. The closest available data report frequencies of body dysmorphic disorder of 4.2–8.8% in the general population and 4.4–12.3% among female students [9]. Similar to societal pressures surrounding the feminine “ideal” of thinness, the masculine “ideal” of being muscular and lean has been linked to rising levels of muscularity-related distress [10]. These concerns may persist even when an individual is objectively muscular and can be associated with social withdrawal, excessive exercise, disordered eating, and increased risk of anabolic steroid use and polysubstance abuse [11]. The use of performance-enhancing drugs, such as growth hormone and testosterone, is a commonly described compensatory behavior and may carry significant psychiatric, hepatic, and cardiovascular risks [12].

Additionally, psychological characteristics such as anxiety, depression, neurosis, and perfectionism have been reported to relate positively to the severity of MD symptoms [13]. Overall, the potential consequences of high muscularity-related distress highlight the importance of identifying individuals with a high symptom burden and possible functional interference. Early recognition of elevated symptoms and associated interference may support timely referral for further assessment and appropriate psychological support, helping to reduce longer-term complications [14]. By conducting this study, we address the scarcity of literature in the Middle East and provide baseline data for future research in Saudi Arabia.

In this study, we hypothesize that elevated MD symptomatology among males is significantly associated with marital status, body mass index (BMI), and educational level. This study aimed to estimate the proportion of gym-going males in Riyadh who screened positive for elevated MD symptomatology. While the use of diverse screening instruments limits direct cross-national comparisons, our findings provide essential insight into local symptom patterns. In addition to estimating the proportion of positive screens, our secondary objectives were to examine the relationship between elevated MD symptomatology and disordered eating symptoms, and to explore associations with sociodemographic factors.

## Methods

### Study design, setting, and target population

This study employed a cross-sectional screening survey design and was conducted in Riyadh, Saudi Arabia. Recruitment took place in commercial multipurpose gyms across Riyadh. The type of gym (for example, bodybuilding-focused, boutique, or general fitness facilities) was not stratified during recruitment. The target population consisted of males aged 18 years or older who were residents of Riyadh and who regularly engaged in gym activities at least twice per week. The inclusion criteria required participants to be male, 18 years of age or older, residents of Riyadh, and regular gym-goers. Exclusion criteria included females, individuals under 18 years of age, nonresidents, and those who did not consistently attend the gym.

### Sample size calculation

The sample size was determined using the Raosoft sample size calculator [15], assuming a 95% confidence level, a 5% margin of error, and a 50% response distribution. Based on the estimated gym-going male population in Riyadh, the required sample size was calculated to be 385 participants [15]. Although 385 responses were initially collected, the final analyzable sample comprised 303 participants after exclusions, representing a 21% shortfall relative to the target sample size. This reduction may have limited the statistical power and precision of multivariable analyses. Therefore, instead of conducting a post-hoc power analysis, we assessed the precision of our estimates by examining the width of 95% confidence intervals (CIs) and the stability of the logistic regression model, treating wide CIs as indicators of reduced precision.

### Recruitment procedure

Participants were approached through general announcements and direct in-person recruitment at commercial multipurpose gyms across Riyadh. Gym type was not stratified at any stage of sampling or recruitment. The questionnaire was electronic, self-administered, and available in both Arabic and English. Before large-scale data collection, a pilot study involving 20 participants was conducted to assess the clarity, acceptability, and structure of the questionnaire.

### Instruments

#### Muscle dysmorphic disorder inventory (MDDI)

The Muscle Dysmorphic Disorder Inventory (MDDI) was used to assess symptoms related to muscularity concerns. An Arabic version of the MDDI, which has demonstrated acceptable internal consistency and construct validity in Arabic-speaking male samples [14], was administered. The MDDI is a 13-item self-report instrument covering three subscales: Drive for Size (DFS), Appearance Intolerance (AI), and Functional Impairment (FI). Items are rated on a five-point Likert scale (1 = “Never” to 5 = “Always”), with higher scores indicating a greater burden of MD-related concerns.

Consistent with prior work in non-clinical gym-going male populations [16], a total score of ≥ 39 was utilized as a commonly accepted screening threshold for elevated MD symptoms. In this study, participants with scores ≥ 39 are classified as screening positive for elevated MD symptoms. This threshold is used to identify individuals at risk of substantial muscularity-related distress and functional impairment in non-clinical populations, rather than to provide a formal clinical diagnostic assessment.

#### Patient Health Questionnaire-Eating Disorders Module (PHQ-ED)

Disordered eating symptoms were assessed using the Arabic version of the Patient Health Questionnaire–Eating Disorders Module (PHQ-ED) [17]. The PHQ-ED is a brief self-report screener that evaluates the presence of binge eating, loss of control, and compensatory behaviors. In this study, a positive screening result was interpreted as indicating possible eating-disorder symptoms that may warrant further clinical assessment; it does not confirm a DSM-5-based diagnosis. Furthermore, as a general tool, the PHQ-ED may underdiagnose muscularity-oriented dietary practices (e.g., rigid “bulking and cutting” cycles) that are common among gym-goers.

#### Demographic data

Participants provided demographic and anthropometric information, including age, height, weight, and field of study. Body mass index (BMI) was calculated as weight in kilograms divided by height in meters squared (kg/m^2).

### Ethical considerations

Before data collection, ethical approval was obtained from the Institutional Review Board Committee (IRB) at AlMaarefa University (Ref. No: IRB24-028). Electronic informed consent was obtained from all participants. Confidentiality and anonymity were maintained throughout the study, and participants retained the right to withdraw at any time without consequence.

### Statistical analysis

Data analysis was conducted using SPSS Statistics for Windows (Statistical Package for the Social Sciences; SPSS), version 26.0. Continuous variables were assessed for normality using the Shapiro–Wilk test. Normally distributed variables were summarized using means and standard deviations (SD), whereas non-normally distributed variables were summarized using medians and interquartile ranges (IQR). For descriptive completeness, both mean (SD) and median (IQR) are reported when informative. Categorical variables were summarized as frequencies and percentages.

Because standard Pearson chi-square (χ²) tests can be unreliable when expected cell counts are low, alternative exact and simulation-based methods were used. For 2 × 2 comparisons, Fisher’s Exact Test was used. For larger contingency tables (e.g., associations between sociodemographic variables and screening-positive status), Chi-square tests with Monte Carlo simulation were used to obtain stable p-values when sparse cells were present. To further support inference accuracy in cases of low expected frequencies, exact and simulation-based approaches were prioritized for sparse tables, including Fisher-type exact testing for larger r×c tables (as appropriate) and Monte Carlo-based Chi-square procedures, to reduce sensitivity to small expected cell counts.

Binary logistic regression using a stepwise procedure was conducted to identify factors associated with screening-positive status for elevated muscle dysmorphia symptomatology (MDDI ≥ 39 vs. < 39). Odds ratios (ORs) with 95% confidence intervals (CIs) were reported. Model fit was evaluated using the Hosmer–Lemeshow test, and statistical significance was set at *p* < 0.05.

## Results

### Demographic characteristics of the participants

In our study, 385 participants completed the online questionnaire. Of 385 responses, 82 were excluded from our analysis due to incomplete data, meeting the exclusion criteria, or incorrect data. Therefore, the study included 303 male participants.

Participants’ ages ranged from 18 to 55 years (mean 26.11, SD 7.24; median 24, IQR 8). Most participants were aged 21–25 years (48.2%), followed by those aged ≤ 20 years (16.8%). The majority were single (75.9%), and most were Saudi nationals (90.4%).

BMI ranged from 16.33 to 49.38 (mean 25.72, SD 5.36; median 25.00, IQR 6.28). Based on BMI categories, 45.9% had a normal BMI, 33.7% were overweight, and 17.2% were obese. Educationally, most participants held a bachelor’s degree (75.6%), while 11.2% had secondary school education or below.

A detailed breakdown of the participants’ sociodemographic and health-related characteristics is presented in Supplementary Table S1.

### Screening results for elevated muscle dysmorphia symptomatology (MDDI ≥ 39) and disordered eating symptoms (PHQ-ED)

Among the 303 participants in the study, 151 (49.8%) screened positive for elevated MD symptoms (MDDI ≥ 39), while 152 (50.2%) screened negative.

Descriptive analysis of individual items revealed notable behavioral and cognitive patterns. For example, 27.1% of the participants reported often wearing loose clothing to hide their bodies, and 28.4% reported usually feeling anxious when missing one or more days of exercise. Additionally, 31.4% of respondents always wished that their arms were stronger. Supplementary Table S2 comprehensively summarizes all 13 item responses, including their frequencies and percentages.

Table 1 summarizes the screening results for MD and eating disorder symptoms, presenting the number and percentage of participants scoring above or below the established thresholds. These results indicate that nearly half of the sample reported elevated MD symptomatology.

Regarding eating disorders, the PHQ-ED screening tool indicated that a small subset of participants (*n* = 5, 1.8%) met the criteria for positive screening, while the vast majority (98.2%) screened negative.

**Table 1:** Screening results for elevated muscle dysmorphia symptomatology (MDDI ≥ 39) and disordered eating symptoms (PHQ-ED)

Screening assessment tool	Results	Count (Column %)
Muscle Dysmorphic Disorder Inventory (MDDI)	Screening-positive	151 (49.8)
	Screening-negative	152 (50.2)
Patient Health Questionnaire–Eating Disorders Module (PHQ-ED)	Screening-positive	5 (1.8)
	Screening-negative	298 (98.2)

This table presents the distribution of responses to two standardized screening tools. A cut-off ≥ 39 on the MDDI is a screening threshold for elevated symptoms in gym-going males and does not represent a clinical diagnosis. For the PHQ-ED, a positive screen reflects endorsement of symptoms on a brief screener and should be interpreted as possible symptoms rather than a confirmed diagnosis

### Association between screening-positive status for elevated muscle dysmorphia symptomatology (MDDI ≥ 39) and disordered eating symptoms (PHQ-ED)

To explore the relationship between disordered eating symptoms and elevated MD symptomatology, a cross-tabulation was performed between participants’ PHQ-ED screening classifications and MDDI screening status (MDDI ≥ 39 vs. < 39). Because one contingency-table cell contained a zero count, a continuity correction was applied to allow estimation of the odds ratio (OR). Fisher’s Exact Test (two-sided) was used to evaluate statistical significance.

The analysis indicated a statistically significant association between screening positive for disordered eating symptoms and screening positive for elevated MD symptomatology (Fisher’s Exact Test, *p* = 0.030). Although the number of participants screening positive on the PHQ-ED was small (*n* = 5), all of these participants also screened positive on the MDDI. Accordingly, participants screening positive for disordered eating symptoms were more likely to screen positive for elevated MD symptomatology than those screening negative on the PHQ-ED. However, the estimated effect size (OR = 11.45) should be interpreted cautiously because it is based on sparse data and a zero cell count (complete separation), which can yield unstable OR estimates (Table 2).

**Table 2:** Association between screening-positive status for elevated muscle dysmorphia symptomatology (MDDI ≥ 39) and disordered eating symptoms (PHQ-ED)

	Eating disorder screening status (PHQ-ED)		*p*-value (Fisher)	OR
	Screening-positive	Screening-negative		
*Muscle dysmorphia screening status (MDDI)*				
Screening-positive	5 (3.30)	146 (96.70)	0.030	11.45
Screening-negative	0 (0.00)	152 (100.00)		

The table summarizes the frequency distribution of participants by eating disorder screening status and MD screening status. Statistical significance was assessed using Fisher’s Exact Test to handle small, expected cell counts. Note: Positive screening reflects endorsement of symptoms on a brief screener and should be interpreted as possible symptoms rather than a confirmed clinical diagnosis. The Odds Ratio (OR) was calculated with continuity correction due to zero cell counts; however, the magnitude should be interpreted with caution given the small number of positive screens. Note: Because one contingency-table cell contained a zero count, the odds ratio (OR) was estimated using the Haldane–Anscombe continuity correction (adding 0.5 to each cell). Fisher’s exact test (two-sided) was used to assess statistical significance

### Association between screening-positive status for elevated muscle dysmorphia symptomatology (MDDI ≥ 39) and sociodemographic variables

An analysis was conducted to examine the association between positive MD screening and various sociodemographic variables. Using the Chi-squared test with a Monte Carlo simulation, significant associations were found with age (*p* < 0.001), marital status (*p* < 0.001), and educational level (*p* = 0.016). No significant associations were found for nationality (*p* = 0.33) or BMI (*p* = 0.37). Table 3 provides a detailed summary of these findings.

Table 3 provides a detailed summary of the associations between MD and sociodemographic characteristics.

**Table 3:** Association between screening-positive status for elevated muscle dysmorphia symptomatology (MDDI ≥ 39) and sociodemographic variables:

Variable	Fisher-Freeman-Halton exact test (*p*-value)	Chi-squared Test with Monte Carlo Simulation (χ²)	Monte Carlo *p*-value
Age	< 0.001	29.69	< 0.001
Marital Status	< 0.001	20.08	< 0.001
Nationality	0.34	0.99	0.33
Educational Level	0.015	10.04	0.016
BMI	0.37	3.19	0.37

The table summarizes the results of statistical tests examining the association between a positive MD screening and various sociodemographic variables. The Fisher-Freeman-Halton exact test, an extension of Fisher’s Exact Test for larger contingency tables, was employed to provide accurate p-values, particularly when expected cell frequencies were minor. Additionally, Pearson’s Chi-squared test with Monte Carlo simulation (10,000 replicates) was used to calculate the chi-squared statistics and corresponding p-values, providing a robust alternative when the classical assumptions of the Chi-squared test are violated. Variables such as Age, Marital Status, and Educational Level showed statistically significant associations with MD, while Nationality and BMI did not

### Predictors of screening-positive status for elevated muscle dysmorphia symptomatology (MDDI ≥ 39): stepwise binary logistic regression

A stepwise binary logistic regression analysis was conducted to identify predictors of screening-positive status for elevated muscle dysmorphia symptomatology (MDDI ≥ 39) among gym-going males. The final model was statistically significant (χ²(7) = 43.761, *p* < 0.001) and showed acceptable fit (Hosmer–Lemeshow *p* = 0.783). The Nagelkerke R² (pseudo-coefficient of determination) was 0.179, indicating that the model explained approximately 17.9% of the variance in screening-positive status.

In the final model (Step 3), marital status emerged as a significant predictor. Compared with single participants, married individuals had significantly greater odds of screening positive (OR = 3.287, 95% CI [1.759, 6.140], *p* < 0.001), and divorced individuals also had significantly greater odds (OR = 20.325, 95% CI [2.320, 178.032], *p* = 0.007). Educational level was significant for graduate-level education; participants with a master’s degree or PhD had higher odds of screening positive than those with secondary school education or below (OR = 13.823, 95% CI [2.597, 73.565], *p* = 0.002). Bachelor’s degree and diploma were not statistically significant predictors (Bachelor’s: OR = 3.823, 95% CI [0.910, 16.060], *p* = 0.067; Diploma: OR = 2.324, 95% CI [0.760, 7.060], *p* = 0.137).

PHQ-ED status could not be evaluated in the multivariable model, and the “Widowed” category was not retained due to sparse data (complete separation/zero cell counts), which yielded non-estimable odds ratios. Estimates for small subgroups—particularly divorced participants and those with graduate-level education—should be interpreted cautiously due to imprecision and model instability. Table 4 presents the final logistic regression model and the identified predictors.

**Table 4:** Predictors of screening-positive status for elevated muscle dysmorphia symptomatology (MDDI ≥ 39): stepwise binary logistic regression

Predictor (Compared to the reference group)	OR	95% CI	*p*-value
*Marital Status (Reference group: Single)*			
Married	3.287	1.759–6.140	< 0.001
Divorced	20.325	2.320–178.032	0.007
*Educational Level (Reference group: Secondary school or below)*			
Bachelor’s Degree	3.823	0.910–16.060	0.067
Diploma	2.324	0.760–7.060	0.137
Graduate Studies (Master’s/PhD)	13.823	2.597–73.565	0.002

This table presents results from a stepwise binary logistic regression predicting screening-positive status for elevated muscle dysmorphia symptomatology (MDDI ≥ 39 vs. < 39). This outcome reflects a questionnaire-based screening classification and does not represent a clinical diagnosis. ORs > 1 indicate increased odds compared with the reference group, whereas ORs < 1 indicate reduced odds

PHQ-ED screening status and the “Widowed” category were not retained in the final model due to sparse data (complete separation/zero cell counts), resulting in non-estimable odds ratios; therefore, the independent association between PHQ-ED screening status and screening-positive MDDI classification could not be assessed in the multivariable model

## Discussion

This study aimed to estimate the proportion of participants screening positive for elevated MD symptoms and to explore the factors associated with these symptoms among male gym-goers in Riyadh, Saudi Arabia. It was found that 49.8% of participants screened positive for elevated MD symptoms using the MDDI. Our findings indicate that nearly half of the participants reported a high symptom burden related to muscularity. It is critical to interpret these results within a continuum-based framework of body image, where symptoms range from normative concerns to significant muscularity-related distress, rather than as fixed clinical diagnoses. Throughout this Discussion, “screening positive” refers to exceeding questionnaire thresholds (MDDI ≥ 39; PHQ-ED positive screen) and is not interpreted as a clinical diagnosis. Screening positive for eating disorder symptoms was also clearly associated with screening positive for MD symptoms. A strong relationship was additionally observed between sociodemographic characteristics (specifically marital status and educational level) and MD symptoms: married and divorced men, as well as men with graduate-level degrees (master’s/PhD), were more likely to report elevated symptoms. BMI was not retained in the final stepwise model, suggesting that muscularity-related dissatisfaction may be more strongly linked to subjective body image perceptions than to measured body size. This pattern is consistent with previous research indicating that men may perceive themselves as “not muscular enough” even when their measured weight or size falls within, or exceeds, the normal range [3, 6, 12, 18, 19].

When interpreting the 49.8% figure, it is essential to consider findings from prior screening-based studies. Reported screening-positive proportions for elevated muscularity-related concerns vary widely across samples, largely because different studies use different instruments and thresholds. Studies using the Muscle Appearance Satisfaction Scale (MASS) have generally reported lower proportions, such as 15.1% of high-risk cases in Hungarian male weightlifters [20] and 17% of at-risk cases in Australian male weightlifters [21]. Studies using measures that emphasize drive for muscularity and functional interference have reported higher values; for example, 61% of Indonesian gym members met an elevated-threshold classification using a drive-for-muscularity approach [8, 22]. Using the MDDI with a ≥ 39 threshold, 37.8% of Turkish gym-going men were classified as at-risk [23]. The current estimate (49.8% screening positive on the MDDI) lies toward the higher end of this range and exceeds the Turkish estimate, supporting the interpretation that between-study variability is strongly influenced by the chosen instrument and cut-off, rather than solely by geography. This figure should therefore be interpreted as reflecting a high level of muscularity-related distress within a specific gym-going subpopulation assessed using a sensitive screening instrument, rather than as a population-level prevalence estimate or evidence of cross-national differences.

Taken together, these findings indicate that differences across countries should not be interpreted solely as cultural differences in the underlying burden of muscularity-related distress. Instead, the observed variability appears to depend heavily on how elevated symptomatology is operationalized in each study: the MASS (dissatisfaction with muscular appearance), the Drive for Muscularity Scale (DMS; drive for muscularity and associated behaviors), versus the MDDI (symptom burden and functional interference). These tools do not use the same thresholds or the same definitions for terms such as “at risk,” “high risk,” or “positive screen.” In other words, reported screening-positive rates and severity estimates are not fixed population constants; they vary depending on the instrument, the cut-off, and how the result is interpreted (e.g., as distress/impairment versus a diagnostic construct). Taken together, these considerations support the interpretation that the 49.8% in our sample reflects a large proportion of gym-going men who are experiencing elevated symptom burden and functional interference related to muscularity, as captured by the screening instrument. However, this proportion does not mean that 49.8% of men in Saudi Arabia meet a confirmed clinical diagnosis. Concerning the relationship between eating disorder symptoms and MD symptoms, our sample showed that, although the number of participants who screened positive for eating disorder symptoms was relatively limited, those individuals were much more likely also to screen positive for elevated MD symptoms.

This pattern is consistent with previous literature on male weightlifters, which describes an overlap between muscularity-focused body image distress, disordered eating patterns, and appearance-enhancing behaviors (including supplement use and structured “cutting/bulking” practices) [8, 21, 22]. These patterns also align with conceptualizations of compulsive exercise and rigid training as emotion-regulation or control strategies, which may co-occur with muscularity-oriented distress without constituting a distinct behavioral addiction or classical eating disorder. In the multivariable model, PHQ-ED status could not be evaluated due to sparse data (complete separation/zero cell counts), yielding non-estimable odds ratios, and BMI was not retained in the final stepwise model. Accordingly, the independent contribution of disordered eating screening status and BMI to screening-positive MDDI classification could not be established in this study. Marital status (particularly being married or divorced) and graduate-level education instead remained strongly associated with elevated MD symptoms. Divorced men showed a higher likelihood of screening positive for elevated MD symptoms compared with single men. However, the magnitude of this estimate should not be over-interpreted due to imprecision and model instability in a small subgroup, likely reflecting the small number of divorced participants and small group size. One possible interpretation is that major life transitions (such as marriage or separation), or perceptions of social and relational competition, may intensify self-monitoring of the body and increase sensitivity to muscular appearance but this hypothesis further requires confirmation through longitudinal research [24]. Similarly, men with graduate-level education (master’s/PhD) showed more than a 13-fold higher likelihood of reporting elevated symptoms compared with men with lower educational attainment. This pattern may reflect the pressures of high performance, perfectionistic traits, and elevated self-expectations in advanced academic or professional environments [8, 25]. These observations suggest that muscularity-related body image distress in men is not limited to “youth gym culture,” but may also be embedded in adult identity, social status, and role expectations.

Interpretation of these findings should also consider the cultural context of Saudi Arabia. Norms surrounding masculinity, physical presentation, and body visibility differ from Western contexts in which most MD measures were developed. Muscularity may function as a symbol of discipline, strength, and self-control rather than solely aesthetic display, and marital status may carry distinct social meanings that influence body-related self-monitoring. These cultural factors may shape how muscularity concerns and self-regulatory behaviors are expressed and reported, underscoring the need for culturally sensitive interpretation and future qualitative research.

Several limitations must be considered. First, this study is cross-sectional; therefore, causal relationships cannot be established. Second, data were collected through self-report screening instruments (the MDDI and PHQ-ED), which are subject to recall bias and social desirability bias. It is also important to emphasize that the MDDI cut-off (≥ 39) is a commonly used screening threshold to indicate “elevated symptoms” among male gym-goers, rather than a formal clinical diagnostic assessment. The reliance on a screening cut-off may overestimate the clinical significance of these symptoms. Many participants screening positive may experience functional impairment in specific areas (e.g., social withdrawal or rigid exercise) without necessarily meeting criteria for a formal diagnosis of muscle dysmorphia based on structured diagnostic interviewing. Thus, the 49.8% figure represents the proportion of individuals who screened positive for a high symptom burden; it does not represent the frequency of confirmed clinical MD in Riyadh.

Third, sampling was restricted to commercial multipurpose gyms in a single metropolitan area, and the type of gym (for example, bodybuilding-oriented, general fitness, or strength training) was not stratified. Additionally, the study sample was predominantly composed of individuals with university-level education, which may reflect selection bias related to gym accessibility or survey participation. This educational skew may limit the generalizability of the findings to all gym-going men in Riyadh, particularly those from less-educated backgrounds. Only males were included in this study as these sampling restrictions limit the generalizability of the findings to women, to other regions of Saudi Arabia, and to more specialized or non-commercial training environments.

Fourth, the primary limitation of this study is the failure to achieve the calculated target sample size. The analysis was conducted on 303 participants, which represents a 21% shortfall from the 385 participants required. This significant reduction in statistical power is the most likely explanation for the instability observed in the logistic regression model. This instability was evident in the very imprecise CIs for several predictors, most notably the ‘Divorced’ category (OR = 20.3, 95% CI [2.320, 178.032]). Furthermore, this sample shortfall directly contributed to the issue of complete separation (zero-cell counts) for sparse categories, which rendered the ORs for Widowed and PHQ-ED status non-estimable. Consequently, while the associations are notable, the results of the regression model must be interpreted with extreme caution and should be considered preliminary. Future studies with larger, adequately powered samples are essential to confirm these findings and provide more stable estimates.

Fifth, anabolic-androgenic steroids (AAS) use and other appearance- or performance-enhancing behaviors were not measured. These factors are recognized internationally and within the Saudi context as being associated with the pursuit of a muscular ideal and body image disturbance. Omitting these variables may conceal important risk factors, mediators, or confounders. Finally, although validated instruments were used, no structured clinical diagnostic interviews were conducted, which means that misclassification is possible for both MD and eating disorders.

These findings point to several important directions for future research. First, there is a need for longitudinal studies to clarify temporal direction: for example, whether significant life changes (such as marriage or separation) and high academic or professional pressure precede and intensify muscularity-related distress, or whether individuals with high baseline body-image distress enter these life stages with a different psychological profile. Second, future work should incorporate structured clinical diagnostic interviews to distinguish between “a positive screening result for elevated symptoms” and “meeting full diagnostic criteria.” This approach would enable a more accurate estimate of the actual diagnostic prevalence. Third, it will be important to measure AAS use and other appearance-/performance-enhancing practices (including current or past use, dosage patterns, and motivations), given their previously reported links with MD, disordered eating, and bulking/cutting behaviors [8, 26]. Fourth, future samples should be broadened to include women, different training subcultures (e.g., bodybuilding, CrossFit, strength training), and multiple regions within the Kingdom to improve generalizability. Fifth, given the high proportion of participants who reported meaningful distress and functional interference related to muscularity, it is reasonable to propose the development of early screening tools and culturally appropriate awareness interventions within gym settings. The aim is not to pathologize exercise or strict dieting in themselves, but rather to identify the point at which training, dietary cycling, or supplement use transitions from a health- or performance-oriented behavior to a behavior driven by compulsive dissatisfaction with one’s body image.

## Conclusion

A high proportion of gym-going men in Riyadh reported elevated symptoms of MD, suggesting substantial body-image distress within this specific population. However, these findings reflect screening results rather than confirmed clinical diagnoses. These results should be viewed through a continuum-based lens of body image distress, emphasizing the need for awareness rather than pathologizing normative fitness behaviors. Further studies employing diagnostic assessments and representative probability sampling are needed to estimate diagnostic prevalence and explore long-term consequences within the Saudi context.

## Supplementary Information

Below is the link to the electronic supplementary material.

Supplementary Material 1

## Data Availability

The datasets used and/or analysed during the current study are available from the corresponding author on reasonable request.

## Abbreviations

AAS: Anabolic-androgenic steroids
AI: Appearance Intolerance (subscale of MDDI)
BDD: Body Dysmorphic Disorder
BMI: Body Mass Index
CI: Confidence Interval
DFS: Drive for Size (subscale of MDDI)
DMS: Drive for Muscularity Scale
DSM-5: Diagnostic and Statistical Manual of Mental Disorders, Fifth Edition
FI: Functional Impairment (subscale of MDDI)
IQR: Interquartile Range
IRB: Institutional Review Board
MASS: Muscle Appearance Satisfaction Scale
MD: Muscle Dysmorphia
MDDI: Muscle Dysmorphic Disorder Inventory
OR: Odds Ratio
PhD: Doctor of Philosophy
PHQ-ED: Patient Health Questionnaire–Eating Disorders Module
R²: Coefficient of determination (pseudo-R²)
SD: Standard Deviation
SPSS: Statistical Package for the Social Sciences
χ²: Chi-square
