# Supplementary material for: Elevated symptoms of muscle dysmorphia and disordered eating among male gym-goers in Riyadh: a cross-sectional screening study
Source: J Eat Disord. 2026 Feb 24;14:71. doi: 10.1186/s40337-026-01556-3 (PMC13040976; doi:10.1186/s40337-026-01556-3)
Supplement: Supplementary file 1 — Supplementary Material 1 [file 40337_2026_1556_MOESM1_ESM.docx]

**Prevalence of Muscle Dysmorphia and Eating Disorder Symptoms Among Male Gym Members in Riyadh, Saudi Arabia: A Cross-Sectional Study**

Khaldoun Ibrahim Marwa^1^, Nawaf Salah Ayad Mohamed ^2^, Hassan Mohammed Abdu^2^, Abdulrahman Abduljabbar Alsarari^2^, Shaden Ibrahim Alsenidi^2^, Nasser Alotaibi^3^, Mohammed Adel Alrehaili^4^, Rayan Saleh Almughyir ^2^, Noof K. Binashikhbubkr^5*^, Anas A. Abdulkader^2^

**^1^** Associate Professor in Psychiatry, Consultant Psychiatrist, AlMaarefa University, Riyadh, Saudi Arabia, **^2^** College of Medicine, AlMaarefa University, Riyadh, Saudi Arabia, **^3^** College of Medicine, Prince Sattam bin Abdulaziz University, Alkharj 11942, Saudi Arabia, **^4^** Jouf University, Al-Jawf, Saudi Arabia, ^5^ College of Medicine and Health Science, Hadhramout University, Hadhramout, Yemen

*Corresponding Author: Noof K. Binashikhbubkr

*Corresponding Email: noufkhalidumar@gmail.com

**Table S1: Sociodemographic and Health Characteristics of the Study Participants**

**Table S2: Frequency and Percentage Distribution of Responses to the 13 Items of the Muscle Dysmorphic Disorder Inventory (MDDI):**

**Table S1: Sociodemographic and Health Characteristics of the Study Participants**

| Sociodemographic and Health Characteristics of the Study Participants | | Count | Column N % |
| --- | --- | --- | --- |
| Age | ≤ 20 years | 51 | 16.8% |
|  | 21–25 | 146 | 48.2% |
|  | 26–30 | 48 | 15.8% |
|  | 31–35 | 23 | 7.6% |
|  | ≥ 36 years | 35 | 11.6% |
| Marital status | Single | 230 | 75.9% |
|  | Married | 61 | 20.1% |
|  | Widowed | 2 | 0.7% |
|  | Divorced | 10 | 3.3% |
| Nationality | Saudi | 274 | 90.4% |
|  | Non-Saudi | 29 | 9.6% |
| BMI | Underweight | 10 | 3.3% |
|  | Normal | 139 | 45.9% |
|  | Overweight | 102 | 33.7% |
|  | Obese | 52 | 17.2% |
| Educational level | Secondary school or below | 34 | 11.2% |
|  | Bachelor | 229 | 75.6% |
|  | Diploma | 19 | 6.3% |
|  | Graduate studies | 21 | 6.9% |

**Legend:** This table presents the distribution of participants according to sociodemographic variables (age, marital status, nationality, and educational level) and health status (as classified by BMI). Age and BMI were categorized for descriptive purposes. Percentages are based on the total sample (N = 303).

**Table S2: Frequency and Percentage Distribution of Responses to the 13 Items of the Muscle Dysmorphic Disorder Inventory (MDDI):**

| Frequency and Percentage Distribution of Responses to the 13 Items of the Muscle Dysmorphic Disorder Inventory | | Count | Column N % |
| --- | --- | --- | --- |
| I think my body is too skinny/slender | Never | 105 | 34.7% |
|  | Rarely | 38 | 12.5% |
|  | Sometimes | 60 | 19.8% |
|  | Often | 47 | 15.5% |
|  | Always | 53 | 17.5% |
| I wear loose clothing so that people can't see my body | Never | 71 | 23.4% |
|  | Rarely | 41 | 13.5% |
|  | Sometimes | 75 | 24.8% |
|  | Often | 82 | 27.1% |
|  | Always | 34 | 11.2% |
| I hate my body | Never | 96 | 31.7% |
|  | Rarely | 36 | 11.9% |
|  | Sometimes | 61 | 20.1% |
|  | Often | 65 | 21.5% |
|  | Always | 45 | 14.9% |
| I wish I could be heavier | Never | 103 | 34.0% |
|  | Rarely | 22 | 7.3% |
|  | Sometimes | 66 | 21.8% |
|  | Often | 70 | 23.1% |
|  | Always | 42 | 13.9% |
| I find my chest to be too small | Never | 105 | 34.7% |
|  | Rarely | 41 | 13.5% |
|  | Sometimes | 64 | 21.1% |
|  | Often | 59 | 19.5% |
|  | Always | 34 | 11.2% |
| I think my legs are too thin | Never | 125 | 41.3% |
|  | Rarely | 39 | 12.9% |
|  | Sometimes | 51 | 16.8% |
|  | Often | 59 | 19.5% |
|  | Always | 29 | 9.6% |
| I feel like I have too much body fat | Never | 40 | 13.2% |
|  | Rarely | 30 | 9.9% |
|  | Sometimes | 85 | 28.1% |
|  | Often | 73 | 24.1% |
|  | Always | 75 | 24.8% |
| I wish my arms were stronger | Never | 31 | 10.2% |
|  | Rarely | 26 | 8.6% |
|  | Sometimes | 58 | 19.1% |
|  | Often | 93 | 30.7% |
|  | Always | 95 | 31.4% |
| I am embarrassed to let people see me without a shirt or t-shirt | Never | 74 | 24.4% |
|  | Rarely | 31 | 10.2% |
|  | Sometimes | 51 | 16.8% |
|  | Often | 81 | 26.7% |
|  | Always | 66 | 21.8% |
| I feel anxious when I miss one or more days of exercise | Never | 46 | 15.2% |
|  | Rarely | 28 | 9.2% |
|  | Sometimes | 74 | 24.4% |
|  | Often | 86 | 28.4% |
|  | Always | 69 | 22.8% |
| I cancel social activities with friends (e.g., watching football, invitations to dinner, going to the movie theater, etc.) because of my workout/exercise schedule | Never | 72 | 23.8% |
|  | Rarely | 55 | 18.2% |
|  | Sometimes | 61 | 20.1% |
|  | Often | 56 | 18.5% |
|  | Always | 59 | 19.5% |
| I feel depressed when I miss one or more days of exercise | Never | 71 | 23.4% |
|  | Rarely | 41 | 13.5% |
|  | Sometimes | 61 | 20.1% |
|  | Often | 69 | 22.8% |
|  | Always | 61 | 20.1% |
| I miss opportunities to meet new people because of my workout schedule | Never | 102 | 33.7% |
|  | Rarely | 39 | 12.9% |
|  | Sometimes | 56 | 18.5% |
|  | Often | 51 | 16.8% |
|  | Always | 55 | 18.2% |

**Legend:** Responses were rated on a 5-point Likert scale ranging from 1 (*Never*) to 5 (*Always*). The table presents both the absolute frequency (n) and the corresponding percentage (%) of participants selecting each response option for all 13 MDDI items. Items reflect three MDDI subscales: Drive for Size (DFS), Appearance Intolerance (AI), and Functional Impairment (FI).
